# Vaginal Seeding: Is There Any Positive Effect in Canine C-Sections?

**DOI:** 10.3390/ani15030416

**Published:** 2025-02-02

**Authors:** Samara Beretta, Renatha Almeida de Araújo, Melissa Oliveira Bianchini, Jaqueline Tamara Bonavina, João Domingos Rocha-Júnior, Nayara Camatta Campos, Lucas José Luduverio Pizauro, Fernanda Andreza Rodrigues-Silva, Gilson Hélio Toniollo, Marita Vedovelli Cardozo, Maricy Apparício

**Affiliations:** 1 Department of Pathology, Reproduction and One Health, School of Agricultural and Veterinary Sciences, São Paulo State University (UNESP), Jaboticabal 14884-900, SP, Brazil; samara.beretta@unesp.br (S.B.); renatha.araujo@unesp.br (R.A.d.A.); melissa.bianchini@unesp.br (M.O.B.); j.rocha@unesp.br (J.D.R.-J.); nayara.camatta@unesp.br (N.C.C.); gilson.toniollo@unesp.br (G.H.T.); marita.vedovelli@unesp.br (M.V.C.); 2 Department of Veterinary Surgery and Animal Reproduction, School of Veterinary Medicine and Animal Science, São Paulo State University (UNESP), Botucatu 18618-681, SP, Brazil; jaqueline.bonavina@unesp.br (J.T.B.); fernanda.rodrigues-silva@unesp.br (F.A.R.-S.); 3 Department of Agricultural and Environmental Sciences, State University of Santa Cruz (UESC), Ilhéus 45662-900, BA, Brazil; lucas.pizauro@unesp.br

**Keywords:** dogs, dysbiosis, microbiology, neonatology, Apgar score

## Abstract

This study aimed to scrutinize variations in the intestinal microbiota of neonatal dogs born through natural birth versus elective cesarean section, focusing on evaluating the influence of vaginal seeding on the microbiota of cesarean-born neonates. Samples were collected from cesarean-sectioned females before anesthesia and from naturally birthing females during prodrome signs, along with neonates at eight time points from birth to 15 days of age. In the cesarean section group, seeding was performed in half of the neonates (cesarean section seeding group; seeding consisted of gently rubbing the gauze, obtained from the mother’s vagina, onto the mouths, faces, and bodies of the newborns), while the other half underwent microbiological sample collection without seeding (cesarean section group). Another group (normal birth group) consisted of naturally born neonates. Microbiota analysis included counting for enterobacteria, *Staphylococcus* spp., and *Streptococcus* spp. The results suggested that vertical transmission played a crucial role, but the method of birth did not emerge as the primary determinant of observed differences. Under study conditions, vaginal seeding failed to effectively modulate the microbiota of neonates born through elective cesarean section. Further investigations into the gut–brain axis are suggested for understanding factors influencing the initial development of the canine intestinal microbiota in neonates born through different delivery routes.

## Simple Summary

Early colonization of the neonatal gut microbiota is vital for immune system development and overall health, as a balanced microbiota supports the maturation of digestive and metabolic systems. Our study investigated whether delivery mode affects the gut microbiota composition in canine neonates, specifically examining the impact of vaginal seeding in those born via elective cesarean section. While vertical transmission was significant, the mode of delivery did not emerge as a primary determinant of microbiota differences, and vaginal seeding was ineffective in modulating the microbiota of cesarean-born neonates. We suggest further research on the gut–brain axis to better understand early microbiota development related to different delivery methods.

## 1. Introduction

Neonatology, encompassing the study of newborn care, physiological aspects, and neonatal conditions, is of paramount importance in veterinary medicine [1]. The neonatal period in dogs corresponds to the first two weeks of life, during which a notable mortality rate persists, primarily attributed to factors such as mishandling and the immaturity of canine fetuses near the end of pregnancy [2,3]. Various non-infectious and infectious causes contribute to neonatal morbidity, including hypoxia, prematurity, hypothermia, hypoglycemia, genetic diseases, trauma, poisoning, viruses, and parasites, with bacterial diseases emerging as particularly significant [4].

Upon birth, the newborn’s gastrointestinal system undergoes significant functional changes, assuming digestive functions previously performed by the placenta. This includes the absorption of essential nutrients crucial for adequate growth and development [5]. The postnatal period witnesses rapid colonization of the newborn’s gastrointestinal tract by microorganisms, a process that is inherently unstable [6].

Initially, it was believed that the mammalian gastrointestinal tract was sterile during intrauterine fetal life, with microorganism introduction occurring postnatally through contact with the mother’s vagina, skin, and ingestion of milk [7]. However, recent molecular techniques challenging this assumption have detected bacteria in the placenta, uterus, or amniotic fluid in various mammals, suggesting potential intrauterine bacterial transmission [8,9,10]. The dynamics of intrauterine bacterial transfer remain a subject of debate [6].

Strains identified in human newborns originating from the maternal microbiota have demonstrated varying adaptability and persistence in the neonate’s gut, emphasizing the intricate nature of neonatal microbial colonization [11]. Conversely, some microorganisms persist in the newborns’ microbiota transiently, suggesting their origin from locations in the maternal body beyond the fecal route, including the back of the tongue, vagina, and skin, without establishing colonization in the neonatal gastrointestinal tract. In contrast, certain more typical fecal species, such as *Bacteroides vulgatus*, *Bifidobacterium longum*, and *Bifidobacterium breve*, endure from birth until at least 4 months of age, indicating their successful colonization of the human infant gut.

The initial composition of gut bacteria plays a pivotal role in influencing the development of the postnatal immune system. In humans, diminished microbial stimulation during childhood is theorized to lead to delayed maturation of the immune system [12]. Additionally, specific intestinal bacteria have been observed to contribute to human health, playing a crucial role in preventing or treating various diseases, such as multiple organ failure, colon cancer, and inflammatory bowel diseases [13].

In recent decades, numerous human studies have linked the rising incidence of autoimmune, infectious, and allergic diseases in both pediatric and adult populations to dysbiosis, signifying an alteration in the normal development of the intestinal microbiota. This dysbiosis is notably observed in children born via cesarean section, who miss exposure to the vaginal microbiota during the natural birthing process.

To address dysbiosis in cesarean-born newborns, researchers have explored vaginal seeding, exposing infants to maternal vaginal fluids. While initial investigations have yielded intriguing results, human studies encounter limitations, particularly the lack of result reproducibility within the same cesarean section, hindering the comparison of individuals exposed to identical conditions. In the realm of veterinary medicine, to date, there are no reports on the potential impact of vaginal seeding on bacterial colonization in the initial days of life.

Consequently, a knowledge gap persists regarding the development of the intestinal microbiota in canine puppies, with this understanding emerging as a fundamental aspect for enhancing the short- and long-term health and well-being of dogs [6]. The mounting evidence implicating the gut microbiome in neonatal health across diverse species underscores the need to identify influencing factors for the overall well-being of offspring, representing a promising avenue of research to decrease morbidity and mortality in the canine species [6].

Recent findings indicating the similarity between the canine and human intestinal microbiota may be attributed, in part, to the domestication of dogs. This process has played a pivotal role in altering the canine intestinal microbiota, resulting in the loss of some bacteria compared to non-domesticated wolves and the emergence of new gastrointestinal bacteria due to interaction with humans [14,15]. The structural and functional similarity of the canine microbiome to that of humans suggests that studies in humans can be predictive of outcomes in dogs, and vice versa, offering a dual benefit. Therefore, dogs, with their multiple births and shared environment, can be deemed valuable experimental models for evaluating the effects of vaginal seeding. In this context, this study aimed to compare the intestinal microbiota of puppies born through natural birth and cesarean section, as well as investigate the potential impact of vaginal seeding in cesarean-born litters.

## 2. Material and Methods

This study followed the recommendations of the Brazilian National Council for the Control of Animal Experimentation (CONCEA) and obtained approval from the Ethics Committee in the Use of Animals (CEUA) at São Paulo State University (UNESP), School of Veterinarian Sciences, Jaboticabal, São Paulo, Brazil (protocol nº 5005/20).

Initially, 33 pregnant dogs were identified, with the intent to allocate half for elective cesarean sections to constitute the cesarean section and seeding group. The remaining half would undergo natural delivery to form the natural delivery group. However, a stringent application of inclusion and exclusion criteria, meticulously implemented to prevent any impact on the initial neonatal microbiota development, led to only 7 female dogs and 28 neonates remaining in this study. 

A total of seven pregnant adult dogs were included in this study, sourced from the clinical routine of our Veterinary Hospital. Among them, three dogs underwent elective cesarean section, while four were monitored during natural birth. The participating dogs, with a body weight ranging from 4 and 15 kg and an age between 2 and 5 years, underwent screening at the Obstetrics and Animal Reproduction Sector (SORA)—UNESP/Jaboticabal. To ensure ethical practice, owners provided their consent for all procedures by signing a “Free Consent Form”, which affirmed their agreement with the clinicians’ decision regarding the appropriate time to perform elective cesarean sections.

The experimental groups were divided as follows: in the cesarean section group, half of the neonates in each litter randomly received vaginal seeding (cesarean seeding group—WS, n = 6 neonates), while the other half underwent only microbiological sample collection without seeding (cesarean group—WOS, n = 6 neonates). Additionally, a third group comprised neonates that were born via natural delivery, and their intestinal microbiota composition was also assessed (natural birth group—AT, n = 16 neonates).

### 2.1. Inclusion Criteria

The bitches included in this study were sourced from breeding kennels located in a controlled environment, where they were in contact only with other animals from the same kennel within an urban area. An ultrasound examination was performed 30 days and 55 days after mating or artificial insemination (according to progesterone concentration; ovulation date), followed by an abdominal radiograph at 55 days to accurately count the fetuses. Only those bitches that presented with four or more fetuses remained in this study. In addition, we included only animals that did not exhibit abnormalities in their blood counts (hemogram) or biochemical levels (creatinine, urea, alanine aminotransferase, and alkaline phosphatase). The cesarean groups consisted exclusively of animals undergoing elective cesarean sections; the optimal timing for a c-section was determined by the clinician based on the criteria outlined in Section 2.3, “screening”.

Newborns included in this study were mandated to consume maternal colostrum within the immunological window of immunoglobulin absorption, adhering to a timeframe of 4 to 6 h post-birth [16]. Those from the same litter were naturally breastfed and cohabitated in the same environment to mitigate the impact of environmental variables on their intestinal microbiota composition. All females, whether undergoing cesarean section or monitored during natural birth, were housed in the same location from day 55 after the initial breeding/artificial insemination and remained there until day 15 after the puppies’ “birth”. Throughout this period, the same individual conducted all evaluations and collections, allowing for consistent daily monitoring within the same environment. This approach ensured that the animals were subjected to consistent environmental and sanitary conditions.

### 2.2. Exclusion Criteria

Female dogs displaying any alterations during pregnancy or identified fetal changes in prenatal ultrasound examinations were excluded from this study. Similarly, those experiencing dystocia (in the normal birth group) or encountering complications during or after cesarean section (cesarean section and seeding cesarean group) were excluded. Additionally, dogs receiving post-coital antibiotics at any stage of pregnancy were not considered.

Regarding newborns, those with an Apgar score below five, identified immediately after delivery, were not included in this study. Furthermore, newborns presenting malformations or defects, such as cleft palate, gastroschisis, anasarca, atresias, and fistulas, among others, were also excluded. Throughout the sample collection period, any newborn exhibiting a condition or developmental change, along with its mother and littermates, was excluded.

For newborns born via elective cesarean section, those experiencing hypoxia at birth (persistently purple mucous membranes, prolonged respiratory difficulty, and need for drug use at the time of neonatal resuscitation) were not included in this study, given that neonatal hypoxia poses a risk factor for the development of early neonatal infections.

### 2.3. Screening

To ascertain the optimal timing for elective cesarean section, ensuring the complete maturity of the puppies, a comprehensive evaluation was conducted, considering the integration of the following factors:

(1) Gestation time. The gestation time, calculated as 58 to 63 days after the first mating or date of artificial inseminations (AI), was determined based on serum progesterone measurement.
(2) Ultrasound assessments. Biparietal diameter measurements [17] were used to estimate gestational age. Additional ultrasound criteria included the assessment of peristalsis presence and fetal heart rate reduction [18].
(3) Reduction in the mother’s rectal temperature [18]. Rectal temperature measurements were taken every 6 h from the 56th day after mating/AI. A reduction of approximately 1–2 °C signaled the estimated onset of parturition within 24 h. Moreover, a comprehensive physical and laboratory examination was conducted, encompassing the heart rate (HR), respiratory rate (*f*), systolic blood pressure (SBP), rectal temperature, blood count, and biochemical profile.
(4) Serum measurement of progesterone concentrations. Progesterone concentrations were measured in serum samples obtained from bitches when signs of fetal maturity were noted on US examination. Progesterone concentration was determined by ELFA (enzyme-linked fluorescence assay) using the miniVIDAS^®^ system (BioMérieux, Marcy-l´Étoile, France) in our laboratory.

### 2.4. Management and Care of Newborns Born by Elective C-Section

Following a comprehensive clinical examination of the genital tract, specific ultrasound evaluations, and confirmation of fetal viability, parturient bitches with an indication for elective cesarean section underwent anesthesia and the subsequent surgical procedure. Anesthesia was induced using 3 mg/kg of propofol (Diprivan^®^, Aspen Pharma indústria farmacêutica, São Paulo, Brazil) for orotracheal intubation, followed by maintenance anesthesia with isoflurane (Isoforine®, Cristália, São Paulo, Brazil). Additionally, the dogs received 2% lidocaine (Xylestesin®, Cristália, São Paulo, Brazil) at a dosage of 2 mg/kg via the epidural route.

During the surgical intervention, the placenta was extracted from the uterus, preserving the cord along with the puppies, without performing umbilical clamping [19]. Subsequently, the neonates received specialized care from a team comprising four to five individuals with experience in neonatal care. Each team member attended to a maximum of three neonates during resuscitation. Immediate neonatal resuscitation commenced upon the exteriorization of the neonate, adhering to the ABC cardiopulmonary resuscitation protocol described by Davidson [20]. The protocol involved airway clearance (A) through meticulous suction using a nasal aspirator, continuous tactile stimulation, and chest compressions to stimulate breathing (B). For neonates with a low heart rate (<80 beats per minute), once breathing was regular, cardiac function (C) was stimulated using sublingual administration of 0.2 mg/kg epinephrine (neonates who required this step were not included in this study). It is crucial to note that newborns with an Apgar score below 5 requiring such interventions were excluded from this study.

Following neonatal resuscitation, the puppies were placed in an incubator set at a temperature of 35–37 °C.

### 2.5. Sample Collection

For females undergoing elective cesarean section, samples from the mouth, vagina, anus, and teats were collected prior to epidural anesthesia (M0) and hair clipping. Sterile gloves and cotton swabs were used during the collection process. In bitches monitored during natural birth, samples from the same regions were obtained when the animals displayed characteristic prodrome signs, such as a reduction in body temperature, restlessness, digging movements, and walking in circles.

Samples from the mouth and anus of newborns were collected using sterile swabs immediately after the rupture of the amniotic membrane (M1). The surgeon and their assistant performed the collection and promptly handed over the swabs to another assistant, who was equipped with sterile gloves and compresses. The newborns were enumerated and identified by birth order, and samples were obtained from the first two and the last two puppies, totaling twelve puppies. Fecal samples from newborns were collected in the first three days after birth and, subsequently, every 3 days for the initial 15 days of life.

In the case of newborns born vaginally, samples from the mouth and anus were collected using sterile swabs after the rupture of the amniotic membrane by the dam. The collection was executed by the assistant wearing sterile gloves and carrying compresses. Similar to the cesarean-born pups, the newborns were enumerated and identified by birth order, and samples were taken from the first two and last two puppies of each litter, totaling sixteen puppies. Fecal samples from these newborns were also collected in the first three days after birth and, subsequently, every 3 days during the initial 15 days of life.

**Figure 1:**
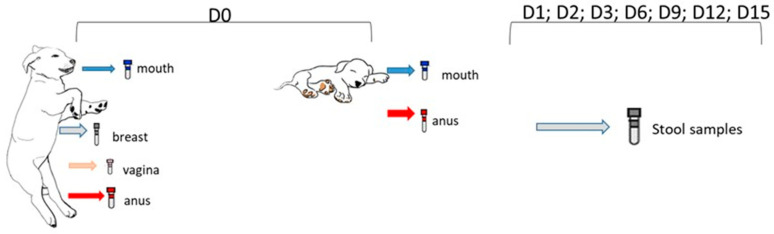
Schematic timeline for sample collection from dams and newborns: “D0” indicates collection during cesarean section or natural birth; “D1” on the first day after delivery; “D2” on the second day; “D3” on the third day; “D6” on the sixth day; and subsequently every 3 days until the 15th day.

Illustrations of the sample collection steps and the specific body parts from which the material was collected are provided in Figure 1. To prepare the smears, the sterile swab’s tip was moved in circular motions on the desired surface. The swab was then placed in a sterile bottle containing 0.1% peptone broth and sent for bacterial counting.

### 2.6. Vaginal Seeding

The procedure for collecting the material for the transfer of vaginal microbiota involved placing sterile gauze in the vagina of the dam during the cesarean section preparation, lasting approximately 40 min. Immediately after the neonates were delivered, samples were collected, followed by vaginal seeding. This seeding process included gently rubbing the gauze, obtained from the mother’s vagina, onto the mouths, faces, and bodies of the newborns. Consequently, seeding was implemented in half of the newborns (seeding group), while the others underwent only the collection of microbiological samples (non-seeding group).

### 2.7. Apgar Score

The Apgar score used in this study adhered to the methodology previously described [21], encompassing the evaluation of the following parameters: the heart rate (HR), assessed with a stethoscope; vocalization, determined through the newborn’s crying; reflex irritability, gauged by gentle manual compression of the limb’s tip; and motility, observed with the neonate in the supination position, taking note of active movements and responses to passive limb movements. Mucosal color was assessed by visualizing the oral mucosa. Each parameter was assigned a score ranging from 0 to 2, and the cumulative score yielded the final Apgar score: 7 to 10 indicated no distress, 4 to 6 signified moderate distress, and 0 to 3 denoted severe distress. The Apgar score was assessed immediately after cardiopulmonary resuscitation and again five minutes later. Neonates who had an Apgar score of less than 5 at this time were excluded from this study.

### 2.8. Microbiological Evaluation

Quantification was performed utilizing the surface plating technique. Each sample underwent an 8-fold dilution in peptone broth (Kasvi, Laboratórios Conde SA, Madrid, Espanha) at a ratio of 1:9. From each dilution, 0.1 mL was used as an inoculum on MacConkey agar (Kasvi, Laboratórios Conde SA, Madrid, Espanha) plates for counting enterobacteria, Mannitol Salt agar (Kasvi, Laboratórios Conde SA, Madrid, Espanha) for *Staphylococcus* sp., and Azida Blood agar (Kasvi, Laboratórios Conde SA, Madrid, Espanha) for *Streptococcus* sp. The inoculum was homogenized using a Drigalski loop, and subsequently, the plates were incubated at 37 °C for 24 h, either in aerobiosis (for enterobacteria and *Staphylococcus*) or anaerobiosis (for *Streptococcus*). Following this incubation period, the colony-forming units (CFUs) were counted, and a calculation was conducted to correct for dilution and area factors as previously described [22].

### 2.9. Statistical Analysis

Microbiological counts were transformed to a logarithmic scale and assessed for normality using the Shapiro–Wilk test. Homogeneity of variances was evaluated with the Levene test. The results were then analyzed using the Kruskal–Wallis test, followed by post hoc comparisons employing the pairwise Wilcoxon test. Statistical significance was considered to be achieved when the *p*-value was less than 0.05.

## 3. Results

### 3.1. Assessment of Maternal Microbiota

We observed no significant difference (*p* < 0.05 using the pairwise Wilcoxon test) in the enterobacteriaceae count between the bitches that underwent normal birth and cesarean section. However, a noteworthy increase in the population of these microorganisms was observed in the vagina of bitches submitted to cesarean section (6.80 ± 5.36 CFU/mL) compared to those of natural birth (2.39 ± 0.00 CFU/mL). Additionally, elevated populations were noted in the anus of bitches from both delivery types (6.63 ± 3.25 CFU/mL in the cesarean and 8.72 ± 4.43 CFU/mL in the natural birth), with teats exhibiting the lowest population of enterobacteria (1.79 ± 1.19 CFU/mL—c-section; 2.39 ± 0.00 CFU/mL—normal delivery).

**Figure 2:**
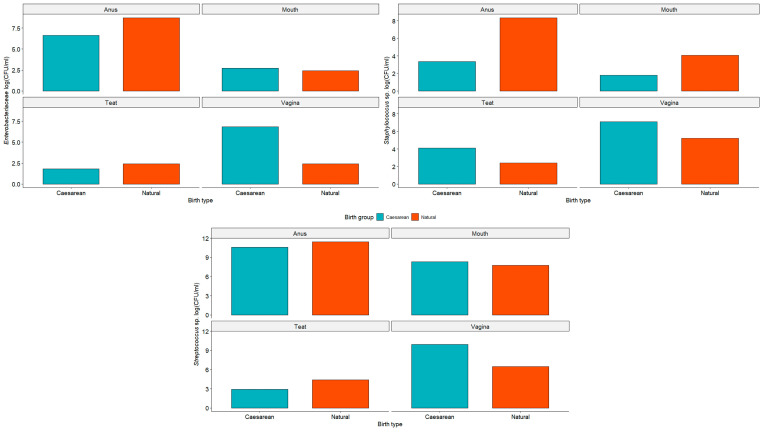
Graphical representation of enterobacteriaceae, *Streptococcus,* and *Staphylococcus* population (CFU/mL) in the anus, mouth, teats, and vagina of bitches that underwent cesarean section and normal birth. No significant difference was observed between the delivery routes (*p* > 0.05) using the Wilcoxon test.

The populations of *Staphylococcus* showed a similar pattern, with high counts found in the anus and vagina. However, a more significant increase in population growth was noted in the mouth and teats, particularly in bitches that underwent natural birth (4.08 ± 2.15 CFU/mL) and cesarean section (4.12 ± 2.23 CFU/mL), respectively (Figure 2). Although no significant difference was found between the delivery type (*p* < 0.05), *Streptococcus*, in general, represented the microorganisms with the largest populations, displaying minimal growth variation between the bitches that underwent cesarean section and natural birth (Figure 2).

### 3.2. Assessment of Neonatal Microbiota

**Figure 3:**
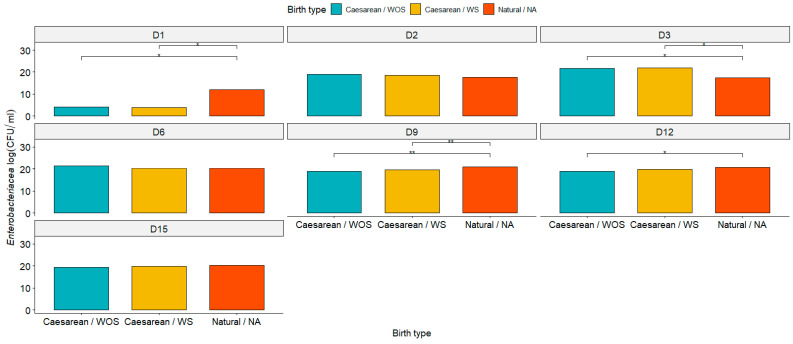
Graphical representation of enterobacteriaceae population on days 1, 2, 3, 6, 9, 12, and 15 postpartum in the cesarean section with seeding (cesarean/WS), cesarean section without seeding (cesarean/WOS), and normal birth (natural/NA). Asterisk (*) and (**): significant differences observed among the groups with *p* < 0.01 and *p* < 0.001, respectively, as determined by the Wilcoxon test.

In general, microorganism populations were low on day 0 (D0) but exhibited a substantial increase on day 1 postpartum (D1). Notably, bacterial populations remained elevated until day 15 postpartum (D15) across all groups (cesarean section without seeding—WOS, cesarean section with seeding—WS, and natural birth—NA). Statistically, there was no significant difference (*p* < 0.05) in the enterobacteriaceae population between the WS and WOS groups, as revealed by the pairwise Wilcoxon test. However, a significant difference (*p* < 0.05) was observed between the WOS and NA groups (Figure 3).

**Figure 4:**
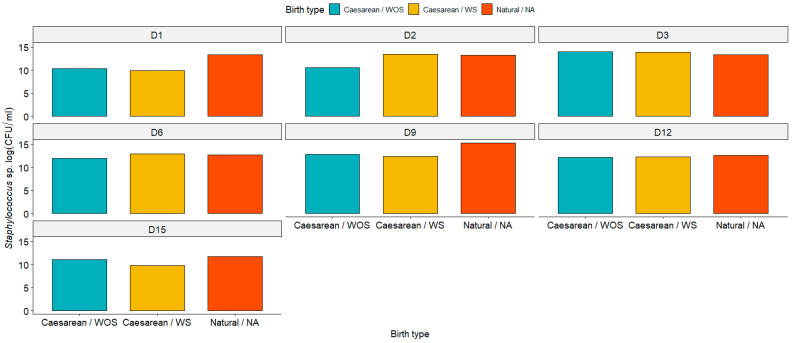
Graphical representation of *Staphylococcus* population on days 1, 2, 3, 6, 9, 12, and 15 postpartum in cesarean section with seeding (cesarean/WS), cesarean section without seeding (cesarean/WOS), and normal birth (natural/NA). No significant difference was observed among the groups (*p* > 0.05) according to the Wilcoxon test.

**Figure 5:**
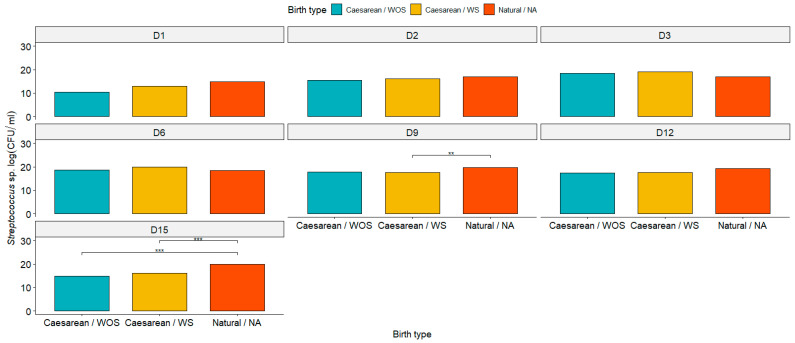
Graphical representation of *Streptococcus* population on days 1, 2, 3, 6, 9, 12, and 15 postpartum in the cesarean section with seeding (cesarean/WS), cesarean section without seeding (cesarean/WOS), and natural birth (natural/NA). Asterisk (**) and (***): significant differences observed among the groups, with *p* < 0.01 and *p* < 0.001, respectively, as determined by the Wilcoxon test.

The *Staphylococcus* population did not exhibit differences (*p* < 0.05) among the groups (Figure 4). Generally, the *Streptococcus* populations followed a similar pattern, showing no differences (*p* < 0.05) among the groups in the initial assessments. However, a significant difference was noted on days 9 and 15 postpartum between the cesarean groups and natural delivery group (Figure 5).

## 4. Discussion

Studies indicate that the development of the intestinal microbiome begins at birth and undergoes transformations across different life stages. In human medicine, it has been reported that most intestinal bacterial strains remain stable for decades [23]. Ferreti et al. [11] identified strains in human newborns that originated from the maternal microbiota, noting that some of these strains are more likely to adapt and persist in the neonatal intestine compared to those not acquired from the mother. On the other hand, some microorganisms were found to persist transiently in the microbiota of neonates, suggesting their origin from maternal body sites other than the fecal route, such as the dorsum of the tongue, vagina, and skin; these did not colonize the neonatal gastrointestinal tract. In contrast, more typical fecal species (e.g., *Bacteroides vulgatus*, *Bifidobacterium longum*, and *Bifidobacterium breve*) persist from birth until at least 4 months of age, indicating successful colonization of the human infant’s intestine. This underscores the importance of early colonization for newborns, as the first established bacteria may potentially shape the host’s intestinal functions for most of their life [24,25]. In veterinary medicine, investigations into the development of the intestinal microbiota in neonates are scarce. In 2017, Guard et al. [6] conducted a study to characterize the fecal microbiome of canine neonates on days 2, 21, 42, and 56 after birth, evaluating the microbiome during neonatal and pediatric development using samples obtained from neonates born only via the vaginal route. Similarly, Garrigues and colleagues [26] collected samples in the first days of life, specifically on days 0, 2, and 7, but only from neonates born through normal delivery, without evaluating the intestinal microbiota of neonates born by cesarean section. In our study, while we did not evaluate the intestinal microbiota during the pediatric period, we assessed the development of the intestinal microbiota in newborns delivered through both natural birth and elective cesarean section throughout the neonatal period (days 1, 2, 3, 6, 9, 12, and 15 post-birth). This marks the first effort to compare the intestinal microbiota development of newborns born via elective cesarean section and natural birth in the canine species.

The composition of the gastrointestinal microbiota is influenced by numerous factors, including age, nutrition, and environment [27,28]. In our study, newborns were mandated to ingest maternal colostrum and were breastfed. Moreover, all females, whether undergoing cesarean section or monitored during natural birth, remained in the same location from day 55 after the first breeding/artificial insemination until day 15 after the birth of the puppies. This ensured uniform environmental and sanitary conditions, aiming to mitigate the impact of “environment” and “nutrition” variables on the intestinal microbiota’s composition. It is well established that the use of medications, such as antibiotics by both mothers and newborns, can disrupt the initial development of the newborn’s intestinal microbiota [29]. Hence, in our study, animals that had any medications were excluded.

According to Sanidad and Zeng (2018) [30], in animal species, the presence of oxygen in the gastrointestinal tract during the first days of life promotes the colonization of obligatory and facultative anaerobes. Oxygen consumption and decreased redox potential (which is positive at birth) play a key role in preparing the intestine for increased colonization of strict anaerobes, later required for healthy intestinal function [6,31,32]. However, in our study, the populations of microorganisms were generally low on day 0 (D0), with a significant increase on day 1 postpartum (D1), including both aerobic microorganisms (enterobacteria and *Staphylococcus*) and facultative anaerobic microorganisms (*Streptococcus*). Garrigues and collaborators [6] suggested that the increase in bacteria abundance in the puppy’s intestine is not only related to oxygen homeostasis but also to the puppy’s milk intake in the neonatal period. This may explain the significant increase on D1 and the sustained high populations until day 15 postpartum (D15) in all groups (cesarean section with seeding, cesarean section without seeding, and natural birth). It is also worth noting that it was initially assumed that the gastrointestinal tract of mammals was sterile during intrauterine fetal life, with microorganism inoculation occurring through contact with the mother’s vagina and skin and the ingestion of milk in the first hours after birth [7]. However, this assessment has been recently contested due to the emergence of molecular techniques that have detected bacteria in the placenta, uterus, or amniotic fluid in different mammals, suggesting the potential transmission of bacteria from mother to fetus in utero [8,9,10]. Despite previous studies that reported low bacterial concentrations and the limitations of culture-based techniques in identifying most organisms, environmental contamination cannot be completely eliminated as a factor when collecting samples from newborns at birth [33,34]. Thus, intrauterine bacterial transfer remains a subject of debate [6], as the techniques used do not fully account for environmental contamination.

The study by Banchi et al. [35] was the first to investigate the feto-maternal microbiota in domestic carnivores using a combination of techniques and rigorous aseptic measures, from the selection of animals to sampling procedures and the inclusion of controls. They suggest that a very low load of bacterial genetic material of unknown viability can be found during healthy full-term pregnancies. Although our study did not investigate the presence of bacteria in the placenta, uterus, or amniotic fluid, the almost negligible bacterial counts on day 0 (D0), with a significant increase on day 1 (D1), may indicate that initial bacterial colonization begins immediately after birth.

After birth, the first step in modulating the intestinal microbiota of the newborn appears to come from vertical transfer from the mother [36]. Saijonmaa-Koulumies and Lloyd [37] state that the meconium of pups after vaginal delivery is colonized by *Staphylococcus* species almost immediately after birth, as this bacterial species is common in the mother’s vaginal microbiota [38]. In the present study, although there were no significant differences (*p* < 0.05) in the enterobacteriaceae count between the bitches that underwent natural birth and cesarean section, there was a greater population increase of these microorganisms in the vagina of bitches submitted to cesarean (6.80 ± 5.36 CFU/mL) compared to the bitches that gave birth naturally (2.39 ± 0.00 CFU/mL). Furthermore, high counts were observed in samples from the anus of bitches from both delivery types, with the teats having the lowest count of enterobacteria. The colonization of enterobacteriaceae appears to come, for the most part, from the maternal anus and vagina, and on days 1 and 9 postpartum, there was a significant difference between the cesarean section (with and without seeding) and natural birth groups, with greater growth in the natural birth group. Since female dogs showed greater growth of enterobacteria in the vagina of the cesarean group and less growth in the vagina of the natural birth group, it is estimated that the predominance of colonization of this bacterial group appears to come from the anus of the female dog. Likewise, a greater population of *Staphylococcus* was observed in the anus and vagina of the evaluated groups, with greater growth in samples from the mouths of bitches that had natural births and in the teats of bitches that had cesarean sections. As there was no significant difference (*p* < 0.05) between the cesarean section and natural birth groups, it was observed that licking and passing bacteria from the maternal teats had less importance in the bacterial colonization of the puppies than the maternal anus and vagina. Thus, our study contrasts with previous studies [33,34], suggesting that the intestinal bacterial colonization of newborns predominantly resembles the maternal vaginal and fecal bacterial composition, regardless of the route of delivery. However, the fact that vertical transmission from mother to offspring plays a decisive role in the formation of the initial composition and diversity of the newborn’s microbiota cannot be excluded. More studies are needed to understand the real cause of differences in the development of intestinal microbiota of canine neonates born by cesarean section and natural birth.

Recent studies on infant microbiota in humans have suggested that the transfer of bacteria from mother to baby is highly dependent on the type of birth, with babies born by cesarean section presenting altered microbiota and, consequently, a greater risk of health problems [39,40]. Similar findings were observed in canine studies that reported lower bacterial diversity in the meconium of puppies born by cesarean section compared to those born vaginally [37,40]. In our study, significant differences were also observed in the population growth of enterobacteria on days 1, 3, 9, and 12 postpartum and of *Streptococcus* on days 9 and 15 postpartum, which were higher in newborns born by natural birth than by elective cesarean section. However, statistically, there was no difference (*p* < 0.05) between the cesarean with seeding and cesarean without seeding groups in the population of bacteria (enterobacteria, *Staphylococcus*, and *Streptococcus*), showing that the use of vaginal seeding was not able to positively modulate the neonatal microbiota of the neonates evaluated in the present study. This suggests, once again, that the route of passage during birth may not significantly interfere with the initial colonization of the neonatal intestinal microbiota, with other factors being involved in the differences in triggering this colonization in natural and cesarean births. Furthermore, our study corroborates the study in humans [41], which concluded that vaginal seeding does not have significant impacts on the intestinal microbiota during the first 2 years of life of babies born via cesarean section. In fact, studies such as Del Carro et al. [42] confirm the extreme importance of maternal microbiota in the formation of the intestinal microbiota of their offspring, showing that each mother has an individual microbiota profile that influences the assembly of the intestinal microbiota of her offspring. These authors suggest that studies consider the modulation of maternal microbiota for the consequent modulation of the intestinal microbiota of neonates. However, Del Carro et al. [43] did not take into account the differences between vaginal delivery and cesarean section, which can influence the initial composition of the neonatal intestinal microbiota. Our suggestion is that hormonal factors involved in triggering normal birth that do not occur in elective cesarean section (since labor is not signaled) may be involved in the differences in bacterial colonization observed between neonates born by natural birth and elective cesarean section. It is already known that there is a bidirectional communication network between the gut microbiota and the brain in humans, which includes neural, endocrine, metabolic, and immunological systems/pathways, known as the gut–brain axis [43,44,45,46]. Endocrinologically, pregnancy in dogs is supported by different mechanisms of hormonal action, controlled through the hypothalamic–pituitary–ovarian axis. The release and participatory balance between different hormones, including progesterone, prolactin, relaxin, and estrogen, guarantee the maintenance and progression of pregnancy. The triggering of labor occurs through the joint action of progesterone and estrogen, as well as oxytocin [47,48]. In elective cesarean sections, especially in humans where a date is set for the procedure prior to the onset of labor, many of the endocrine mechanisms are not activated. Therefore, new studies related to the intestine–brain axis suggested that there may be differences involved in the development of the intestinal microbiota of newborns born by elective cesarean section, both in humans and animals.

Although our study represents an initial investigation into the development of the neonatal intestinal microbiota in canine neonates born through normal birth and elective cesarean section, as well as the use of vaginal seeding to modulate the initial microbiota of neonates born via elective cesarean section, several limitations should be noted.

First, the number of animals used in this study was not ideal. Our sample size did not reach the desired number, as many animals initially included had to be excluded to ensure the reliability of this study. Factors such as nutrition, environment, use of antibiotics, comorbidities, and other variables that can interfere with the development of the neonatal intestinal microbiota were carefully considered. Consequently, the number of females and their newborns was reduced from an initial 33 females and 132 newborns to 7 females and 28 newborns. Another limitation was the absence of DNA sequencing using metagenomics, which was not performed due to financial constraints. The bacterial groups selected for colony-forming unit (CFU) counts were chosen for their importance and prevalence in the microbiota of the selected samples. While DNA sequencing provides valuable information about microbial diversity, it can be limited in terms of genus-level identification. Additionally, CFU counting was prioritized to evaluate bacterial cell viability, which was crucial for addressing our hypothesis.

## 5. Conclusions

The present study is the first in the canine species to compare the initial development of the intestinal microbiota of neonates born through cesarean section and those delivered naturally.

Key findings indicate that while vertical transmission from mother to offspring plays an important role in the initial development of the canine intestinal microbiota, the route of birth does not appear to be the primary determinant of the observed differences between newborns born via natural birth and cesarean section. Furthermore, the use of vaginal seeding did not prove effective in modulating the initial intestinal microbiota development in neonates born through elective cesarean section.

Future studies should be considered to better understand the fundamental factors contributing to variations in the development of the intestinal microbiota in dogs born through elective cesarean section versus natural birth. Exploring the gut–brain axis may offer valuable insights into the endocrine and hormonal aspects influencing the composition of the neonatal intestinal microbiota in different types of birth (natural and cesarean section).

## Data Availability

The original contributions presented in this study are included in the article. Further inquiries can be directed to the corresponding author.
